# *Ich kann nicht anders:* Social Heroism as Nonselfsacrificial Practical Necessity

**DOI:** 10.3389/fpsyg.2018.02269

**Published:** 2018-11-20

**Authors:** Bryan Smyth

**Affiliations:** Department of Philosophy and Religion, University of Mississippi, Oxford, MS, United States

**Keywords:** heroism, practical necessity, phenomenology of the body, embodied action, pre-reflective intentionality, self-sacrifice, habitus

## Abstract

Most self-reports of heroic action in both reactive and social (proactive) cases describe the experience as involving a kind of necessity. This seems intuitively sound, but it makes it unclear why heroism is accorded strong approbation. To resolve this, I show that the necessity involved in heroism is a nonselfsacrificial practical necessity. (1) Approaching the intentional structure of human action from the perspective of embodiment, focusing especially on the predispositionality of pre-reflective skill, I develop a phenomenological interpretation of Bernard Williams' notion of “practical necessity” as an endogenous existential necessity. (2) I then offer a view of reactive heroism as instantiating this kind of necessity by literally embodying certain socially affirmed values in a way that is not self-sacrificial. This evinces a deep social bond, and it is this bond, rather than the action itself, that is the ground of approbation. (3) I then discuss how this construal of reactive heroism can be extended to cases of social heroism by way of a necessity that is internal to the agent's individual character. Similarly to reactive cases, a social hero literally embodies a certain ethical commitment such that her actions are likewise instances of nonselfsacrificial practical necessity. (4) I then discuss how the commitment perceived in cases of social heroism pertains to the actualization of “surplus validity,” such that whereas the reactive hero is praised for embodying shared value, the social hero is praised for embodying a commitment to actualizing the concrete potential of such value more fully The approbation accorded to social heroism is therefore tied inextricably to a normative judgment concerning such immanent progressive transformation.

Hier stehe ich, ich kann nicht anders! Gott helfe mir, Amen!

Here I stand, I cannot do otherwise! May God help me, Amen!

These famous words are conventionally attributed to Martin Luther speaking in his own defense on 18 April 1521 at the Diet of Worms against accusations of heresy. While as a matter of historical fact it is unlikely that he uttered anything in this exact form^1^, it is widely held that this attribution nonetheless reflects quite accurately the spirit of his oratory that day. Refusing steadfastly to recant his published views that were highly critical of the papacy and other institutions and doctrines of the Catholic Church, views that had already led to his excommunication by Pope Leo X, Luther bravely stuck by his convictions and knowingly risked severe personal consequences. As the Edict of Worms, issued shortly thereafter by Emperor Charles V, expressed the expected official outcome, Luther was to be apprehended, captured, and punished as “a notorious, obstinate heretic,” all forms of sympathy or support for whom were also harshly proscribed in no uncertain terms.

As it turned out, however, the Edict's measures against Luther went largely unenforced, and he managed to live and to develop his reformational views for another quarter-century. Be that as it may, the dramatic words attributed to him on that day in Worms—“Here I stand, I cannot do otherwise!”—are often taken as emblematic of the uncompromising fortitude characteristic of what is nowadays termed “social heroism,” *viz*., cases of *proactive* heroic action that typically unfold over a long period of time and with a great deal of reflection, and which aim at preserving or instituting social values (Franco et al., 2011, p. 100f)^2^—as opposed to *reactive* or “split-second” cases that involve actions occurring at a particular moment and seemingly without any reflection at all^3^. Unlike typical reactive cases, social heroism generally excludes immediate threats, physical and otherwise, to the agent's well-being, although it does usually involve significant on-going hardships and long-term risks, up to and possibly including the risk of death^4^. Such differences notwithstanding, however, in the same way as reactive heroes often report after the fact that they plunged into their actions more or less spontaneously, without any of the sort of self-conscious deliberation that would constitute their heroic actions as truly *optional*—saying, for example, “I just did what *had* to be done,” the self-descriptions offered by social heroes of their conduct often likewise involve something effectively equivalent to Luther's dictum of necessity (as I shall call it), i.e., the “I cannot do otherwise!”—saying, for example, “I *could not* stand by and do nothing”^5^.

Characterizing both reactive and social heroism as involving some kind of necessity may be entirely appropriate, at least at a rhetorical level. Yet upon scrutiny it is somewhat puzzling. For while we may be intuitively inclined to accord considerable praise and esteem to a hero so described, it is not altogether clear *why* we would do so, if we take it as being indeed literally the case that she was unable to do otherwise and thus arguably not morally responsible for the specific action or course of actions in question. To be sure, we might still hold a positive view—as we would, for example, in a reactive case of someone whose uncontrollable flatulence happened to incapacitate an active shooter. But our response would surely differ, in both nature and degree, from a case in which someone—like James Shaw, to take a recent example—who achieved the same result through direct physical intervention that evinced “selfless disregard for his own safety”^6^. Or similarly, while we might hold a positive view of someone who felt internally compelled to engage wholeheartedly in a selfless course of risky prosocial action, even if we believed that her felt compulsion to do so stemmed ultimately from an exogenous process of political indoctrination, this would again surely differ in both nature and degree from a case in which someone conducted herself in the same way, yet of whom we believed the felt compulsion to do so was in fact a genuinely internal or endogenous factor. At the same time, however, it also seems intuitively compelling that the strong approbation accorded in cases of either reactive or social heroism is directly tied to the real possibility that the agent in question *could have* acted otherwise—more specifically, to the real possibility that the individual could simply have *not* engaged in the action or actions in question and instead remained, like most others, a bystander. Assuming *arguendo* the absence of this possibility in cases of heroic action, it becomes unexpectedly unclear just why we would hold these individuals in any higher regard than those who perform equivalent actions but from a physical or otherwise exogenous kind of necessity.

As with our intuitions concerning most everything that concerns us, those with regard to heroism are generally fairly reliable (e.g., when and where to accord heroic approbation), but they are also potentially subject to serious confusions (e.g., concerning the nature and grounds of such approbation). In particular, most intuitions concerning heroism are informed by a widely (if implicitly) held but, I think, ultimately incorrect set of assumptions to the effect that heroic action is a *moral* phenomenon—specifically, that it is morally supererogatory (i.e., goes above and beyond the call of moral duty)—and that as such it is to be praised on account of the self-sacrifice it implies on the part of the individual agent. For *moral* praise—the kind of praise one is owed when one does one's duty, or, more relevantly here, when one engages in supererogatory action—is premised implicitly on the notion that such action involves the self-conscious subordination of personal inclination in favor of an impartial or altruistic moral imperative. It implies, in other words, that moral action is freely chosen over equally possible but more self-regarding options, that in this sense moral action—and supererogatory action especially—is essentially self-sacrificial, and that it is considered praiseworthy precisely for this reason. Here, I submit, is the source of the puzzle regarding heroism that is self-reported by the individual agents themselves as involving a kind of internal compulsion. For taking them at their word, if they really could not do otherwise, then there is no sense in speaking of them as having made a choice on that occasion, let alone a self-sacrificial choice (cf. Archer, 2015, p. 119)^7^. If that is so, then there would seem to be no grounds for moral praise—and lacking any alternative conception of approbation, there would thus seem to be no grounds for any praise at all (although we certainly may hold a positive view). Their action would be seen as a natural event that simply happened fortuitously, like a bolt of lightning (or flatulence) that fells an active shooter.

Now, for the sake of the present argument I shall assume that heroism in general—at least pending certain conceptual clarifications—does in fact instantiate a certain form of necessity, and in particular that Luther's dictum of necessity does indeed apply to social heroism. But I want to show that this is not at all inconsistent with the relatively high degree of positive approbation that is normally accorded to it. To show the possibility of maintaining both of these intuitions in the face of the puzzling situation sketched out above, I propose to rethink the sense of necessity that heroism involves, especially cases of social heroism, in order to clarify the underlying nature of heroic action and on this basis to suggest a new understanding of the normative grounds of heroic approbation in general.

The analysis will be developed across four main steps:

1. Approaching the intentional structure of human action from the perspective of embodiment, focusing especially on the predispositionality of pre-reflective skill, I first develop a phenomenological interpretation of Bernard Williams' notion of “practical necessity” as an endogenous existential necessity or incapacity to do otherwise, and consider this in connection with ethical action in particular.
2. I then offer a view of reactive heroism as instantiating this kind of necessity by *literally* embodying certain socially affirmed universal values in a way that is not self-sacrificial and therefore not, properly speaking, moral, nor *a fortiori* supererogatory. In terms of approbation, then, it follows that when we praise a reactive hero, we are not so much praising what she *does* as what she *is*, to wit, her predispositional corporeal being as it is summoned and activated, so to speak, by the particular situation. In spontaneously crystallizing an important shared social value in a situation in which most others fail to act, her heroic action evinces a deep bond with her social world, and it is this bond, I suggest, rather than the action itself that brings it to light (although that is not unimportant), that awes us and elicits approbation. We praise the reactive hero, in other words, because in realizing *herself*, she gives powerful expression to who *we* are^8^.
3. I then discuss how this corporeal construal of reactive heroism as positively self-realizing rather than self-sacrificial can be extended to cases of social heroism. Here I show that the necessity to which Luther's dictum alludes can be understood as a constraint internal to the embodied, pre-reflective intentional structure of the proactivity in question, such that the idea of doing fundamentally otherwise would thus imply self-abnegation on the part of the agent far more clearly than in reactive cases. For while much tactical deliberation may occur with regard to specific actions, the social hero's reflection on her overall goal is not a matter of considering alternative possibilities, but of self-discovery with regard to the necessity that is internal to and hence deeply expressive of her individual identity or character. Similarly to reactive cases, then, a social hero literally embodies a certain ethical commitment such that her actions are likewise carried by normatively valenced vectors of pre-reflective intentionality and are equally instances of nonselfsacrificial practical necessity^9^.
4. Turning to the question of approbation, I discuss how, unlike reactive cases, which evince the spontaneous affirmation of an established social value, the commitment perceived in cases of social heroism has more to do with the actualization of what I shall call, borrowing a term from Axel Honneth, “surplus validity,” that is, the fact that the meaning and scope of a given society's existing ethical norms can be altered and expanded. Thus, whereas the reactive hero is praised for embodying shared value, (rather than for her action *per se* or the manner of its performance), the social hero is analogously praised for embodying a commitment to actualizing or instituting the concrete potential of such value more fully. The approbation accorded to her is therefore tied inextricably to one's normative judgment concerning this immanent progressive transformation. If we praise a social hero, then, it is because in and through realizing herself, she gives concrete expression, not so much to *who we are*, as with reactive heroism, but to *who we are aspiring to be*^10^.

## Embodied action and practical necessity

As I have discussed in more detail elsewhere (Smyth, 2010, 2014), the reasoning here draws its initial inspiration from Maurice Merleau-Ponty's phenomenology of embodiment (Merleau-Ponty, 1945). In particular, it draws on the claim that while mind–body dualism (Cartesian or otherwise) is certainly false, concretely understood our embodied existence does involve a certain temporal duality that we may express using the spatial metaphor of levels: there is a “habitual” level (linked to the past), and an “actual” level (tied to the present). Each of these levels is orthogonal to the distinction that can be drawn analytically between mind and body. Generally speaking, the actual level pertains to personal existence or “ipseity” in the sense of occurrent bodily and reflective intentional states, in relation to which the habitual level forms the impersonal or pre-personal background. The latter is the anonymous accretion of internalized—or, to use a suggestive phenomenological metaphor, *sedimented*—experiences that develops dynamically across time and which, in establishing certain pre-reflectively intentional habitualities, transforms the psychosomatically integral organism in enduring and intrinsic ways, and provides the enabling and constraining conditions for personal existence at the actual level.

We might initially think of this in terms of the sedimentation of perceptual and motor experiences, and take the development through repeated practice of specific skills that emphasize such experiences and their coordination—for example, learning to ride a bicycle or to swim, to play racquetball or the clarinet—as paradigmatic of the sort of habitualities involved. But setting habitual compulsive disorders aside, we must bear in mind that even with the most routine of habits, the contexts in which they are repeatedly enacted are never exactly the same—indeed, it is characteristic of highly developed skills, or skillful “expertise,” to be sensitive to situational differences and thus correspondingly flexible or improvisational (Dreyfus and Dreyfus, 1980; Dreyfus, 2004). Even the actualization of perceptual and motor habitualities, then, is never a matter of sheer automaticity, but rather of increasingly strong predispositions that are always subject to and coordinated with other situational factors.

These predispositions are thus not as simple or straightforward as they may at first seem. To begin, they are situationally transposable—a racquetball player is not an absolute beginner in her first squash match, a clarinetist shifts to the oboe more easily than a cellist. And this transposability could be much less plain to see—running a daycare for special needs children might equip one with skills well-suited to chairing an academic department. One can thus do something for the very first time yet still be habitually predisposed to it. In other words, what I am calling habitual predispositions can, in terms of their underlying intentionality, be quite general with regard to the situations in which they operate—they need not manifest as an overtly regular pattern of behavior, nor arise from such a pattern. And they can also operate negatively and unselfconsciously, as when, for example, someone phobicly avoids crowded or constrained spaces due to a repressed traumatic experience. In all these cases, what we are pointing to in the habitual level of embodied existence are pre-reflective intentionalities that are situationally-responsive, and which operate predispositionally as the anonymous background conditions that give personal existence its idiosyncratic profile.

But the operation of these habitualities is not to be observed solely in terms of idiosyncrasies. For it can be seen no less clearly in terms of the mannerisms, postural schemata, modes of comportment, and speech patterns, for example—what Marcel Mauss (1936) called “body techniques”—that more broadly form culturally common corporeal “styles” or “idioms” (Goffman, 1971; Elias, 2000[1939]). Even when considered (as I am doing here) in prediscursive embodied terms, perceptual and motor idiosyncrasies develop in specific social, cultural, and historical contexts. The habitual level of embodied existence thus internalizes—or *incorporates*—and hence comes quite literally to incarnate certain aspects of the individual's intersubjective and social milieux. This does not mean that everyone is alike—within a given socio-cultural context there are typically differences, for example, pertaining—sometimes quite problematically—to perceived phenotypical characteristics. In a dynamically aggregated or intercorporeal sense, then, habitual embodiment is thus the primary and central locus of what Pierre Bourdieu (in particular) termed “*habitus*,” pithily glossing this as the “durably installed generative principle of regulated improvisation” (Bourdieu, 1977, p. 78), under the dispositional auspices of which individual perception and behavior unfold.

This model of embodied existence as involving an actual level that in general does not coincide with a socially embedded habitual level suggests conceiving selfhood as a function of the ongoing tension between the two levels—a tension which can but certainly need not be experienced negatively, and it suggests conceiving self-realization as the process of negotiating this tension and striving to bring about an optimal or at least minimally disharmonious integration of the two dimensions. Logically, this can be pursued in two broad ways, either of which could represent a path to authenticity (if we wish to speak that way): one can reflectively cast one's intentions forward with the aim of modifying the habitus—that is, of pushing the envelope of “regulated improvisation”—by engaging in actions directly toward which one does not currently have strong predispositions; or else one can refrain from any such reflective projection and fall back on the pre-reflective intentionalities residing already in the habitus. One can, so to speak, resolve to break out of a perceived rut, or else go with the flow. In either case, the options are wide. But looked at in this “existential” way that excludes any purely biological reality, it is worth noting that optimal self-realization need not place any overriding priority on biological self-preservation—one can try to break out of a rut by trekking solo across the Sahara, for example, or taking up alligator wrestling, while one could go with the flow by jumping on the fentanyl bandwagon, or by heeding a patriotic recruitment campaign and enlisting for armed conflict. Needless to say, existential ruts and flows are highly situationally contingent.

Actions pertaining to the ethical life of society exemplify self-realization along these lines with particular clarity. For in general, at any given moment, there is usually a discrepancy between our actual personal inclinations and the morally praiseworthy actions that are normatively expected of us—indeed, as noted earlier, such actions are morally praiseworthy primarily because of the altruism that they demand. But it is situationally contingent as to which dimension (if either) of our embodied being is the more other-regarding. For example, one might live in a social context in which it is considered normal and acceptable to walk past a hungry homeless person with indifference—in such a case, falling back on the habitus, going with the flow, would seem to be the ethically deficient path, and that a morally superior response would require an overridingly deliberate, reflective effort to offer alms, food, or other assistance. Conversely, one might live in a society in which such a manner of response was itself a predispositional feature of the habitus, that is, of one's habitual embodiment, such that even if on some particular occasion one happened to be very hungry oneself or otherwise self-concerned, the habitual disposition might still hold sway^11^. In short, inasmuch as we do actually fulfill our ethical expectations, in some cases the intentional structure of our action stems from a process of reflective moral deliberation, while in other cases any such reflection is eschewed and we act simply on the basis of habitual pre-reflective intentionalities. Sometimes we choose to do good, sometimes we just do it.

The former scenario may be more familiar when thinking about ethical issues, and it may garner more scholarly attention. Yet it is arguably more exceptional than typical. To be sure, situations that prompt reflective moral deliberation do arise, but on the whole it seems that more of what goes on in human coexistence corresponds to the latter scenario, in which one falls back upon what we might call one's “pre-reflective ethical know-how” (DeSouza, 2013), the specific kind of “skillful coping” or “ethical expertise” that guides us through our “everyday ongoing ethical coping” (Dreyfus and Dreyfus, 1991, 2004) in ways that are strongly analogous to how other forms of acquired skillful expertise, like bicycle riding or clarinet playing, likewise guide us through everyday coping in their relevant contexts. Comprising “the interrelated, and possibly conflicting, values, virtues, goods, rights, behaviors, attitudes, etc. which make up the ethical fabric” of one's social context (DeSouza, 2013, p. 284), such pre-reflective familiarity with the contours of that context's normative landscape or *ethical habitus* involves a tacit perceptual sensitivity to the axiological import (e.g., the recognition of the intrinsic value of humanity) that gives rise to situations of ethical significance in the first place. As a kind of “ethical second nature” that we come to embody (literally) through socialization and interpersonal experience, it guides most of our quotidian interactions with others, while also providing the motivational and evaluative background for any more explicitly formulated moral intentions.

What is especially important to note here is that as with the skills involved in bicycle riding or clarinet playing, “expert ethical comportment” is profoundly spontaneous in that as a kind of pre-reflective know-how, it involves no explicit deliberation. Consider the following phenomenological observations made several decades ago by Maurice Mandelbaum concerning actions that are spontaneous yet have a situationally-sensitive ethical import:

I sense the embarrassment of a person, and turn the conversation aside; I see a child in danger and catch hold of its hand; I hear a crash and become alert to help. Actions such as these (of which our daily lives are in no small measure composed) do not, at the time, seem to spring from the self: in such cases I am reacting directly and spontaneously to what confronts me. […] In such cases it is appropriate to speak of ‘reactions’ and ‘responses’, for in them no sense of initiative or feeling of responsibility is present (1955, p. 48).

As Mandelbaum went on to observe in a striking fashion, from the perspective of such actions themselves, “we can only say that we acted as we did because the situation *extorted* that action from us” (Mandelbaum, 1955, p. 49, italics added). From the first-person perspective of the agent, in other words, there is no experience of choice but rather of a situational necessity that elicits a certain response from oneself^12^.

This view of necessity offers a compelling phenomenological interpretation of what Bernard Williams had in mind with the notion of “moral incapacity”—“the kind of incapacity that is in question when we say of someone, usually in commendation of him, that he could not act or was not capable of acting in certain ways” (Williams, 1993, p. 59)—and how this leads to a “practical necessity” (Williams, 1981), in cases where one is thusly incapable of performing any action *but one*, typically in the form of being incapable of *not* performing *that one*, however the modal situation may appear to third-person observers. For his part, Williams viewed this situation in terms of character, noting, for example, that such incapacities and necessities can be constitutive of one's character, and that “to be an expression of character is perhaps the most substantial way in which an action can be one's own” (1981, p. 130). As Kyle Fruh expressed it more recently, “[t]he issuances of practical necessity are to be seen not as constraints imposed on an agent, but as expressions of the core characteristics of the agent” (2017, p. 32). My own aim here is to show that character in this regard is best understood phenomenologically in the pre-reflective terms of habitual embodied action. Agentive incapacities, no less than capacities, are grounded here, and it is in this way, specifically in terms of corporeal sedimentation of the ethical habitus, that we can make best sense of how ethical principles can be “internalized and appropriated as part of one's identity” (Schlenker et al., 2009, p. 319), such that the individual is spontaneously able to enact them appropriately, and to do so in an endogenously necessary way that is fully self-realizing—as Colby and Damon said of the moral exemplars they studied, “[n]one saw their moral choices as an exercise in self-sacrifice” (Colby and Damon, 1994, p. 300, italics removed)^13^. It is in terms of the two-level phenomenological model of embodiment, in other words, that we can best make sense of how an ethical imperative could manifest as a nonselfsacrificial practical necessity.

The key point here is that the strength of this necessity correlates—somewhat counterintuitively—with the degree of “ethical expertise,” that is, with the degree to which one's ethical actions are unreflectively spontaneous—as with bicycles and clarinets, *higher* degrees of ethical virtuosity go hand in hand with *lower* degrees of reflective deliberation. In such situations, “[o]ne feels that one's comportment was caused by the perceived conditions […] We do not experience our intentions as causing our bodily movements; rather, in skillful coping we experience the situation as drawing the movements out of us” (Dreyfus, 2002, p. 379f). Now, to describe ethical action as being in any way caused from without is potentially misleading. For the idea is just that the actions in question are matters of pre-reflective intentionality which, rooted deeply in our corporeality, is something (not unlike muscular tonus, say) that we do not normally experience in subjective terms. So while we may say that the situation elicits or draws the action out of an agent, it is nonetheless *her* response to it. The point is that there is such a close and intimate attunement to the situation, such a profoundly immersive oneness between it and the agent's habitual embodiment, that like a steeply banked turn at the velodrome or a complex musical phrase in the orchestra pit, there is really only one possible action to take—and there is no sense at all that the necessity of this action is external to the agent or that it in any way threatens or compromises her agency. In a very concrete sense, then, in such situations agents make a virtue out of necessity—and in ethical situations, this sense is doubly literal, with virtue and necessity going hand in hand.

## Reactive heroism as embodied phenomenon

Having laid all that out, I will now turn to reactive heroism and relate it to this framework. In doing so, I shall take as a possible example the case of Tom Lee (1885–1952), an unskilled African-American laborer who, although unable to swim, used his small boat to pull 32 people from the Mississippi river when the sternwheeler *M.E. Norman* overturned near Memphis on May 8, 1925. Although his actions received extraordinary commendation from the public and from government officials, Lee himself expressed the sentiment of personal disavowal that is characteristic of heroic action in analogous cases: “I guess I didn't do any more than anyone else would have done in my place” (Finger, 2014)^14^.

I submit that cases of reactive heroism like this are matters of embodied habitual action. On the face of it, it may seem highly implausible to claim that an action like that of Tom Lee on that day in 1925 is “habitual,” since he had never done anything quite like it prior to that occasion, and never did anything quite like it again. There is absolutely nothing routine about the actions in question. But a key point from the phenomenological discussion above (section Embodied Action and Practical Necessity) is that the nature of habitual action does not lie essentially in manifest patterns, but rather in the prior corporeal internalization of certain perceptual and motor skills as pre-reflective intentionalities which then, in the form of predispositional schemata, enable individuals to act in a spontaneously “expert” way in an indefinite number of different situations^15^. So it is immaterial that most heroic actions, including Lee's, appear to be unique one-off occurrences. With regard to Lee, the idea would be that he had internalized certain universal features of the ethical habitus of his society, such as the impartial recognition of the intrinsic value of humanity; that these features had “sedimented” in the habitual dimension of his embodied existence such as to equip him with the pre-reflective ethical know-how that predisposed him to do what he did on that day, and to do so under the kind of practical necessity that accompanies spontaneous “expert” behavior in general^16^. Indeed, in cases like this, where someone acts in an ethically extraordinary *yet clearly spontaneous* way, how else could we make sense of it? If we do not interpret the action as the practically necessary actualization of habitual predispositions, then we would have no choice but to dismiss it as a fortuitous fluke to which no particularly strong approbation would be due.

To be sure, talk of “ethical expertise” can seem odd, and it is potentially misleading if it suggests a high level of cognitive engagement. As discussed above, though, the point is exactly the opposite—in the sense in which it is being used here, “expertise” describes a degree of skill development at which, even while remaining situationally responsive, the need for explicit reflective deliberation is precisely obviated. As Colby and Damon said of the moral exemplars they studied, “we saw no ‘eking out’ of moral acts through intricate, tortuous cognitive processing. Instead, we saw an unhesitating will to act, a disavowal of fear and doubt, and a simplicity of moral response” (Colby and Damon, 1994, p. 70)^17^. And this may apply well to cases like that of Tom Lee. More generally, reactive heroism is characterized by the eschewal of moral reflection in favor, so to speak, of “going with the flow”—understood here as falling back into a state of unselfconscious immersion in one's habitual predispositions, something that may well be a necessary condition of the “optimal psychological functioning” often referred to precisely as “flow” (cf. Csikszentmihalyi, 1990, p. 218; Annas, 2008). In this context of expert ethical spontaneity, the reactive hero does not *choose* to do the right thing, but *just does it* as a matter of the practical necessity established by her own characteristic predispositions in the particular circumstances of the situation^18^. So while it may appear that reactive heroism involves prosocial self-sacrifice on the part of the agent, I submit that a more coherent and compelling understanding would view the action in question as subjectively incarnating and identifying with universal features of the ethical habitus, and as being a nonselfsacrificial matter of authentic self-realization for this reason^19^.

It is therefore the fact that the reactive hero is *someone who does not need to deliberate*—that in her very being ipseity and habituality momentarily coincide—that grounds the approbation that we accord. It is not the action *per se*, since after all this is done on the basis of a practical necessity—it is rather the fact of its being endogenously necessary that truly awes us. Likewise, there is nothing intrinsically estimable about going with the flow and acting on the basis of habitual predispositions—no doubt some of the white Americans that Lee saved were especially prone to engage in objectionably racist behavior (by *our* lights) whenever they fell back on *their* habitual predispositions. What is so highly commendable about reactive heroism is the fact that the hero is someone who is ontologically predisposed to do good, a fact that is brought to light by the unusual nature of the heroic action, but which is not identical with it nor reducible to it. What awes us about the reactive hero, then, is the recognition that on account of having internalized features of the shared habitus, she gives extraordinary expression to an ordinariness we share, and which is partly constitutive of our sociality—our approbation celebrates an existential or even *intercorporeal* continuity with the hero that is structurally absent from cases of moral supererogation^20^.

## Social heroism and practical necessity

I shall now turn to consider whether the above account of reactive heroism as a phenomenon of embodied action that instantiates nonselfsacrificial practical necessity can be extended to cases of social heroism^21^. A key difference here is that reactive heroism, owing to how its spontaneity precludes reflection, does not admit of a contrasting scenario in which the intentional structure of an outwardly equivalent action would involve reflective deliberation rather than habitual predisposition. With social heroism, however, there is such a contrast with scenarios of moral supererogation in which outwardly equivalent actions are performed electively in preference to at least one less altruistic alternative—i.e., cases in which there is no necessity (practical or otherwise) involved, hence “I *could* do otherwise!”—and which as such *are* self-sacrificial^22^. There is nothing wrong with this, of course, and it generally merits high moral praise. But it differs fundamentally from social heroism, even if on the face of it the actions in question are indistinguishable, inasmuch as the social hero, like her reactive counterpart, literally embodies the relevant social value, whereas the supererogatory action is reflectively mediated.

Here I shall take as a possible example the case of Virginia Foster Durr (1903–1999), who, despite being born into a life of Southern white privilege, devoted most of her adult life to the struggle for equal civil rights in the United States, in particular with regard to effecting desegregation and outlawing the poll tax in the South (see Colby and Damon, 1994, pp. 92–133; Durr, 2003). Concerning her activism, Durr expressed views that are fairly typical of social heroism. With regard to abolishing the poll tax, for example, “I thought the right to vote was something that everybody ought to have,” and she considered this to be an established norm that was just not yet fully instituted. “Although I seemed radical to other people, and was considered radical, I never thought of myself as being radical because I was simply doing what was common everywhere else.” With regard to racial desegregation, she claimed that “there were no choices to make.” There were, of course, choices to make, but these did not bear upon the basic project, which was shot through with a kind of necessity: “as far as the decisions I made concerning my part […] in the racial struggle in the South, it wasn't a decision, it was something that grew over a period of years and one thing led to another.” In short: “I did what I felt I had to do” (Colby and Damon, 1994: 133, pp. 121, 71, 120, 124). My aim here is to sketch out how Luther's dictum of necessity could apply to cases like this^23^.

As discussed above (section Embodied Action and Practical Necessity), the idea of practical necessity drawn from Williams helps to make initial sense of the necessity that people like Durr report in their experience, by articulating how it may be conceived in terms of ethical constraints and incapacities rooted internally within the agent's character. For this is how the experience of necessity can be rendered consistent with their no less robust experience of free agency. But what we also saw is that character in this sense is best and most concretely understood in phenomenological terms, that is, in terms of the pre-reflective intentionalities that make up the habitual predispositionality of our embodied existence. For this affords the most compelling model of how values, principles, and any other elements of the ethical habitus can be internalized so as to become stable and irreducible features of an individual's character—how, in other words, the “integration of agency and communion” that is characteristic of moral exemplars is actually achieved (cf. Frimer et al., 2011). In cases of reactive heroism this allows us to understand how dramatic instances of seemingly self-sacrificial ethical behavior can occur in a completely spontaneous way. In cases of social heroism, conversely, it will allow us to understand the perseverance, the recurrently reaffirmed motivation, and the longitudinal continuity that characterize the agent's endeavors over time. In both sorts of cases, though, the basic point is that it is literally true and not merely a suggestive piece of rhetoric to say that heroism is the embodiment of ethical commitment.

This is perhaps best approached by way of the well-known problem of the “judgment–action gap” (Straughan, 1986), *viz*., the fact that at a cognitive level the vast majority of people tend to affirm various ethical judgments that they consistently fail to act on. This is something that is shown with particular (if simplified) clarity whenever a bystander applauds a social hero—she approves, so why did she remain a bystander? This problem has motivated much recent work that tries to steer away from an exclusive focus on cognitive factors toward more “personological” views that emphasize the role of character in bridging this gap. My own point is that we just need to push this line of reasoning farther: if the spirit is willing, so to speak, but the flesh is weak, then it stands to reason that we should direct our attention to our flesh, to our corporeality, and to how it serves as the site of mediation between judgment and action.

In the same way as a reactive hero, in perceiving a situation in a certain way, manifests an embodied incapacity to be a disengaged bystander, that is, acts under an endogenous form of nonselfsacrificial practical necessity, so too the social hero, in perceiving a state of affairs in a certain way, can be understood as manifesting a similar incapacity with an analogous necessity. It differs, though, in that in this case the necessity is not that of a spontaneous and unreflective “expert” response, but of an increasingly recognized visceral refusal to do otherwise. It is in this sense that social heroism may be said to involve personal growth and a process of reflective self-discovery. While after the fact the reactive hero may certainly learn something about herself and grow personally as a result, this dynamic is internal to the protracted experience of the social hero, where it can even involve developmental change at the habitual level—this is certainly true in the case of someone like Durr. It is thus central to the reaffirmation of the social hero's motivation that she must make as events unfold. To be clear, though, within the intentional structure of her proactive project, this reflection is not a matter of deliberating among logically possible alternatives, but rather of discovering that, for her, there really is no acceptable alternative. Much more clearly than in reactive cases, then, doing fundamentally otherwise here would be existentially self-abnegating on the part of the agent. Although the social hero still goes with the flow in the sense that her actions are ultimately carried by normatively valenced vectors of pre-reflective intentionality in essentially the same way as in reactive cases, this typically occurs with a much higher level of self-awareness and personal investment.

The reason why the judgment–action gap applies to most of us is that while we may approve and endorse the actions of a social hero through our considered ethical judgments, it typically appears to us that in order to act on those judgments, what would be required of *us* would be an act of supererogation—hence a self-sacrificial act—and we consequently experience a debilitating hesitation and unwillingness. This is why most of us fail to act (and why we misperceive heroes as engaging in supererogatory action), at least on those judgments that exceed the moral baseline (i.e., the minimum threshold that is expected of us in a given social context, which most of us have internalized more or less successfully). Now, some *do* manage to rise above the tension and bridge the gap by engaging in supererogatory action. But in the conceptual scheme that I am trying to work out here, I want to identify such cases as categorically distinct from heroism (see Smyth, 2018). For it makes a world of difference whether the intentional roots of an ethical action are reflective or pre-reflective—whether it is enacted negatively through a reflective decision to engage in personal self-sacrifice for moral reasons, or positively as a matter of self-realization that coincides with an internalized universality. The reason why most of us might approve of a social hero's actions yet be unable to follow suit is simply because they have something we lack—which, for the sake of that simplicity, I will just call a “heroic body.” It is important to bear in mind that this refers to the way in which bodily existence incorporates psychosocial aspects of moral culture so as to thereby become the locus of a hybrid kind of “*biopsychosocial* resilience,” rather than just a natural matter of biology^24^. There is much that might be said about this (see especially Efthimiou, 2017). But for present purposes the point I wish to make is just that as with reactive cases, what is impressive and commendable about social heroism is that it makes a virtue out of an endogenous necessity, and that the locus of this necessity is habitual corporeality. Social heroism emerges from an existential “*must*” at the habitual level of embodiment, rather than a moral “*ought*” at the actual level, and so it is precisely on account of having a heroic body—a body that “stands” a certain way (as in “Here I stand”)—that Luther's dictum applies to cases of social heroism.

But this does not yet fully clarify the question concerning approbation, to which I now turn.

## Social heroism and social progress

Heroism in general is a matter of actualizing “sedimented” ethical universality under an endogenous practical necessity, hence in a nonselfsacrificial way. But there is a further distinction between reactive and social cases that should be noted here. Whereas reactive cases enact norms that are already recognized and affirmed in an effectively universal way within a given social context (e.g., innocent lives should be saved), but do so spontaneously in situations (e.g., dangerous ones) in which the judgment–action gap is especially acute, cases of social heroism typically have to do with establishing or instituting new ethical norms (e.g., civil rights should be equal for all). I have argued that, in both sorts of cases, the relevant heroic actions are ultimately carried by vectors of embodied pre-reflective intentionality, i.e., going with the flow, rather than reflective decisions. Yet social heroism can, and often does, cut against the grain of society—it can (and often does) encounter disapproval or opposition in ways that reactive heroism seldom does. This may seem puzzling—how can such disapproval or opposition occur when one is going with the flow of the sedimented ethical habitus? Assuming *arguendo* a unified habitus^25^, if one is just reflecting social norms back after having internalized them, then how could there be any such tension?

To answer this question we must clarify that social heroism does not involve instituting ethical norms that are utterly novel. As with Virginia Durr, it is rather a matter of recognizing and trying to redress the fact that certain socially existing norms – e.g., individual civil rights, or the right to vote—while valid and valuable in themselves, are not instituted correctly or completely according to their intrinsic sense. To give a name to this, I shall borrow the term “surplus validity” from Axel Honneth's theory of social recognition (Honneth, 2003, p. 186). This refers to the fact that, within a given social context, the meaning and scope of existing ethical norms can, through social and cultural struggle, be altered and expanded. This is the normative space that makes an “immanent critique” of society possible, that is, a critique of society that bases itself normatively not upon some arbitrary or ideal criteria pulled out of thin air, but upon the very norms of the society itself. The point of such a critique is precisely to show that these norms are not fully actualized, that this entails some form of injustice, and to point toward the kind of social progress that might make good on the shortcoming. So even though there is common ground—evinced by the shared value that reactive heroism embodies—different views of the meaning and scope of these norms are often contested—hence the possibility of disapproval or opposition to social heroism.

As I have described it here, social heroism as an embodied phenomenon is located entirely within the normative space of surplus validity. It has nothing to do with idealism in the loose pie-in-the-sky sense of ungrounded moral aspirations. There may be a place for that—in social contexts in which the ethical habitus is very rudimentary or thoroughly compromised with evil, for example, there may be no possibility of a viable immanent critique. And hence no heroism. For just as surplus validity is what makes immanent critique possible, by the same token *it is the basis upon which there could possibly be an embodied commitment to a socially transformative goal*^26^. Reactive heroism is not socially transformative. More generally, a society maintains itself when individuals act morally, i.e., when they do their ethical duty. But it does not progress. There's nothing wrong with acting morally, of course, and in wanting others to do likewise—the inherent self-sacrifice is minimal, and the benefits usually outweigh it. But if we are concerned with progressive social transformation, morality comes up disappointingly short. For all it can offer is a view of supererogation in which self-sacrifice is ratcheted up considerably, with the result that the judgment–action gap opens up much more widely. That supererogatory actions are often generally praised is evidence of broad commitment to values that transcend the minimum moral baseline of society, but the judgment–action gap is a formidable obstacle to progress pursued in this way.

If we are concerned with social progress, and if we reject as unrealistic any extraneous normativity, then our focus should be on realizing surplus validity. And in terms of action there would seem to be two broad ways to approach that—moral supererogation and social heroism—which reflect the two-level model of embodied existence with which we began. The point I wish to make here is simply that the approach of social heroism affords grounds for greater optimism with regard to the prospects of realizing surplus validity. For in contrast to moral supererogation, it indicates a way of bridging the judgment–action gap that it is much more plausible to believe could be cultivated on a broad scale. For if within a given ethical habitus there are indeed latent but nonetheless real tendencies that would exceed the moral baseline of the *status quo*, then it seems entirely reasonable to suppose that they could be realized or instituted concretely much more effectively to the extent that individuals' commitment to them was a positive matter of their self-realization through existential identification, even to the point of becoming a practical necessity, rather than as a moral aspiration to be achieved through self-sacrificial supererogation^27^.

In sum, regardless of how we may construe it intuitively, our approbation of social heroism is an expression of our normative judgment concerning this kind of immanent social progress, and of our recognition that the hero's self-realization is bound to the historical transformation it implies. Whereas the reactive hero is praised relatively uncontroversially for embodying shared value, the social hero is praised, more contentiously, for embodying a commitment to actualizing the concrete potential of such value more fully. If we do in fact praise a social hero, then, it is because we sense that in and through realizing herself, she gives powerful concrete expression, not so much to *who we are*, as with reactive heroism, nor to who we might ideally aspire to be, as with moral supererogation, but to *who we are in fact aspiring to be*^28^.

## Author contributions

The author confirms being the sole contributor of this work and has approved it for publication.

### Conflict of interest statement

The author declares that the research was conducted in the absence of any commercial or financial relationships that could be construed as a potential conflict of interest.

## Footnotes

^1^These words themselves were not in the original verbatim transcript, but were added to the record later—see, for example, (Bainton (1950), p. 185); (Atkinson (1981), p. 161f); (Brecht (1985), pp. 460, 537 note 24); (Wilson (2007), pp. 153, 170).

^2^This in no way implies that Luther himself is necessarily to be regarded or celebrated as a hero. While some Protestant Christians might so regard him (presumably overlooking, among other things, his virulent anti-Semitism), most devout Catholics probably do not (although they might regard Thomas More, say, or Jeanne d'Arc in analogous ways), and many more people might not care at all one way or the other about any of these religious figures. All of this can thus be taken as illustrating the social relativity of attributions of heroism, and of the specific sort of approbation that goes with such attributions. Nevertheless, it is possible to study the phenomena of heroism scientifically, and the present analysis is intended as a conceptual clarification that can help to undergird such study.

^3^Although I shall use the more conventional expression “social heroism,” the key contrast with reactive cases is better captured with the term “proactive,” which would imply as a corollary the difference in durational character. In any case, none of this should be taken as implying that reactive or ‘split-second’ heroism is not also *social* in some important sense (in a similar way that social heroism, like anything proactive, is at the same time also *reactive* in a certain sense).

^4^Although Luther himself may have been prepared to die for his actions, there is no essential connection between the sentiment he is taken to have expressed and the idea or ideal of martyrdom (cf. Kendrick, 2010, pp. 24–49), where Luther's words are taken as emblematic of a martyrial conception of heroism). In general, according to the view that I shall develop below, while heroism excludes any overriding concern with biological self-preservation, it is inconsistent with any genuine martyrial aspirations.

^5^Philip Zimbardo may have expressed himself hyperbolically in claiming that “[e]very single person who's identified as a hero always says, ‘How could I not do it?”’ (Sommers, 2009, p. 59), but it is a seemingly constant feature of various kinds of closely studied cases, many examples of which can be found in Berkowitz (1987), Oliner and Oliner (1988), Colby and Damon (1994), Monroe (1996), and Paldiel (2000), among others. However, even if, with regard to human action in general, first-personal reports have some degree of epistemic privilege, they are not authoritative and cannot on their own play any explanatory role with regard to the intentional structure of an agent's actions. In this paper, I will take putative heroes' self-reports of a sort of necessity simply as an invitation to explore whether something like that could possibly be true, not as evidence that it *is* true.

^6^See Nashville municipal resolution RS2018-1182 https://www.nashville.gov/Metro-Clerk/Legislative/Resolutions/Details/abe66859-36d1-4908-80fa-aae1d66950a2/2015-2019/RS2018-1182.aspx

^7^I have discussed the nonselfsacrificial nature of heroism more fully in Smyth (2018) in the context of drawing out the contrast within the category of (what I'll call) “extraordinary prosocial action” between heroic and supererogatory action, in which the latter is, while the former is not, a matter of self-sacrifice. The key point is that supererogatory (or “saintly”) action is strictly speaking *moral* action in that it issues ultimately from processes of reflective moral deliberation, with the implication that the agent *could* do otherwise, while “heroic” action is *not* moral in this sense, in that it issues predominantly from embodied habituality, and as such involves the sort of predispositional necessity discussed here. This is a crucial distinction, but one that is typically glossed over. This distinction is central to my overall approach, but in the present paper I do not discuss “saintly” action directly. In what follows, then, it should be borne in mind that I am considering heroism to be a *sub-category* of extraordinary prosocial action, and that as such I am *not* claiming that all such actions are heroic in the sense that I shall ascribe to this term (i.e., the other sub-category of supererogatory action remains). As I note below, this may entail upsetting some existing intuitions, but the hope is that on the whole the resulting view will be able to avoid many of the ambiguities and equivocations that still affect contemporary thinking about heroism.

^8^Critical attention must always be paid to the casual way in which first-person plural pronouns are used in philosophy and the human sciences. Here, “we” merely denotes some particular community as a reflection of the fact that attributions of heroism are not universal—it is well known that what is lauded as heroism by one community may be vilified by another—and that they hinge upon a multitude of cultural, historical, and political variables. But as noted above, the phenomena of heroism can still be the object of scientific study.

^9^The expression “pre-reflective intentionality” denotes those aspects of one's intentional relatedness to the world that fall below the level of explicit self-conscious awareness. It includes the kind of intentionality that is involved in, for example, basic activities like movement or grasping, forms of habitual skill, the ways in which one responds to and orients oneself in a situation, one's lived sense of space and time, and the ways in which one tacitly carries elements of the past while projecting horizons of anticipation.

^10^It is worth noting here that although the reconceptualization of heroism that I develop below may upset some of our existing intuitions, its goal is to uphold the most important ones by gaining a firmer handle on the relevant phenomena, so as to be able to solve the puzzle concerning the strong approbation that heroism typically elicits. It is also worth emphasizing that this rethinking of heroism unfolds in a conceptual space that temporarily places in abeyance any empirical research concerning heroism that has been conducted on different conceptual bases. Such research can speak neither for nor against what I'm doing, because at least for the time being the meaning of heroism is precisely in question.

^11^For the purposes of this conceptual analysis, these sort of homespun thought experiments can be sufficient. To carry the discussion further, however, would of course require corroboration from credible empirical research. But it is crucial to bear in mind that all empirical research is conducted within certain conceptual and theoretical frameworks, and that if one wishes, as I do, to offer an alternative framework, then special care must be taken when trying to assimilate the results of existing research.

^12^Cf. Merleau-Ponty: “The body is but one element in the system of the subject and his world, and the task elicits from him the necessary movements through a sort of attraction at a distance, just as the phenomenal forces at work in my visual field elicit from me, without any calculation, the motor reactions that will establish the optimum equilibrium between them, or as the customs of our social setting or the arrangement of our listeners immediately elicits from us the appropriate words, attitudes, and tone” (1945, p. 123f). Various echoes of this general perspective can also be found in Varela (1999), Wright (2007), Rietveld (2013), among others.

^13^To reiterate an earlier point, there is nothing authoritative about such self-reports, mediated or otherwise. But they can play an instructively illuminating role, and it is in that sense alone that they are being invoked here.

^14^In taking Lee as a possible example of my view of reactive heroism, what I wish to do is simply take the manifest bio-historical facts of the case—people were caught in the river, Lee was in the vicinity, and he responded in a seemingly spontaneous and selfless way—and see if an interpretation of it in terms of embodied habitual action can plausibly fit those facts. The example itself is not being called upon to do any theoretical work, and I am not taking his statement—which at any rate is vague and uninformative with regard to the intentional structure of his actions—as in any way authoritative.

^15^This may be seen as a phenomenological specification of the idea of “special training” as discussed by Kohen et al. (2017, p. 10f).

^16^To reiterate, this is a possible interpretation of the Lee case, *any* account of which would be equally interpretive, inasmuch as the relevant details concerning Lee's intentional state at the time are empirically inaccessible, as they typically are even in contemporary cases. Competing interpretations would have to be adjudicated on the basis of their relative plausibility, for example, and their coherence with other commitments.

^17^See note 13 above.

^18^While some connections may be drawn with discussions of automaticity (cf. Snow, 2010; Lapsley and Narvaez, 2014; Rees and Webber, 2014), I would caution against applying that notion to cases of heroism, lest we confound existing intuitions concerning approbation. The main salience of disclosing phenomenologically the habitual level is to locate a meaningful ground intermediate to reflective deliberation and automaticity on which to base an understanding that captures both the ethical content of heroic action and its spontaneity, and that can therefore do justice to our intuitions concerning both of these features of heroism, even if it upsets others.

^19^There are some clear similarities between this view and the main tenets of virtue ethics, which is similarly concerned with dispositional character rather than reflectively intentional actions as such. But I would caution that such affinities could easily be overstated, at least inasmuch as virtue ethics typically retains a morally other-regarding focus that still valorizes self-sacrifice in a vestigial way, whereas heroism implies a more expressly positive account of agential self-realization (cf. Franco et al., 2016).

^20^Strictly speaking, if supererogation implies reflection, then there cannot be cases of spontaneous supererogation. In part this reflects confused intuitions concerning reactive heroism, but there may be cases falling between reactive and social heroism—see next note.

^21^Clearly this distinction is somewhat simplistic in that there may be cases falling between the spontaneity of reactive heroism and the protractedness of social heroism, and which might not exhibit practical necessity. But for present purposes I shall leave this possibility aside.

^22^This is what in a previous work (Smyth, 2018) I refer to as “saints”—see note 7 above.

^23^As with Tom Lee above, I am not claiming that the case of Durr, and her self-reports in particular, provides any direct evidence for my claims. Rather, I just want to hang my interpretation on the bio-historical facts of the case as a way to illustrate its plausibility—neither example is being called upon to do any theoretical or conceptual work.

^24^It is not just a matter of moral culture, but other acquired skills and habitualities too. Cf. (Kohen et al. (2017), p. 2): “The real reason that most people are bystanders rather than heroes is that most people are out of practice.”

^25^This assumption may be false, but if so, then the problem can be solved much more easily.

^26^This is why heroism as I have conceived it is of interest to critical social theory. This is a connection I shall develop more fully in subsequent work.

^27^Although this raises myriad questions concerning how it could actually be brought about, it is along these lines alone, I submit, that heroism could possibly be “banal” in the egalitarian sense described by Franco and Zimbardo (2006), and how individuals in general are, as Zimbardo puts it, “heroes in waiting” (see Sommers, 2009).

^28^I would like to thank Olivia Efthimiou and the two referees for their helpful comments on an earlier version of this paper.
